# Material selection for tooth-supported single crowns—a survey among dentists in Germany

**DOI:** 10.1007/s00784-020-03363-9

**Published:** 2020-06-16

**Authors:** Angelika Rauch, Annett Schrock, Oliver Schierz, Sebastian Hahnel

**Affiliations:** 1 Department of Prosthetic Dentistry and Dental Materials Science, University of Leipzig, Liebigstr. 10, Haus 1, 04103 Leipzig, Germany; 2 Institute for Medical Informatics, Statistics and Epidemiology (IMISE), University of Leipzig, Härtelstr. 16-18, 04107 Leipzig, Germany

**Keywords:** Ceramic, Composite resins, Dental alloys, Dental material, Dental restoration (permanent), Survey

## Abstract

**Objectives:**

This study aimed to survey dentists in Germany to identify their favored materials for the fabrication of tooth-supported single crowns (SCs) depending on the location of the abutment teeth and the preparation margin.

**Materials and methods:**

The survey included questions regarding demographic characteristics of dentists/their dental practice and preferred restorative materials for the fabrication of SCs for abutment teeth 16, 11, 34, and 36 with either supra- or subgingival preparation margins.

**Results:**

Between August 2019 and February 2020, 721 dentists participated in the survey; responses from 33 dentists were excluded from data analysis because the dentists did not work in Germany or provided less than one fixed dental prosthesis/month. Dentists favored ceramic materials independent of the location of the abutment tooth and preparation margin (56.6–92.2%). CAD/CAM resin composites or full metals were preferred by only a few participants. A significantly higher proportion of dentists recommended porcelain fused to metal for subgingival preparation margins than for supragingival margins (*p* < 0.001). Characteristics of dentists/dental practices influenced a single scenario (11 subgingival) that was dependent on the dentist’s time since graduation. When asked to specify the ceramic materials, numerous participants wrote a free response (5.7–7.8%) or did not answer (0.7–4.8%).

**Conclusions:**

Dentists in Germany selected restorative materials for SCs depending on the clinical scenario. Since numerous dentists did not specify the ceramic materials, postgraduate information and education might help to extend expertise.

**Clinical relevance:**

The results of this survey provide insight into the favored materials of dentists for the fabrication of tooth-supported SCs.

## Introduction

Dentists providing restorative and prosthetic treatment are confronted with a vast and steadily increasing variety of dental materials that are available on the market. Precious and base metal alloys have been available for decades. Consequently, evidence-based statements can be given regarding their long-term performance in fixed prosthodontics [1]. However, while fixed prosthetic restorations fabricated from alloys are still the standard treatment option in the posterior dentition in formalities of German national health care insurance companies, these restorations do not meet the patients’ expectations associated with favorable esthetic appearances. While partial or complete veneering of alloy restorations (i.e., porcelain fused to metal, PFM) can relevantly improve esthetic appearance, shortcomings such as gray shimmering or complications such as chipping of the veneer are frequently described [2, 3]. With regard to this aspect, the use of tooth-colored materials might be helpful to overcome some of these problems. However, especially for recently introduced materials such as translucent zirconia, resin composites, or some lithium-X-silicate ceramics [4] fabricated with computer-aided design/computer-aided manufacturing (CAD/CAM) techniques, clinical outcomes in view of quality and longevity are sparse or missing [5, 6]. Moreover, current and future advances in digital dentistry can lead to the implementation of novel fabrication techniques and materials in daily dental practice that might serve as easy-to-use tooth-colored alternatives in fixed prosthodontics [7–9].

The restorative materials that are currently available on the market feature a wide range of mechanical properties that have an impact on the indication spectrum, adequate preparation design, and appropriate luting methods. Regarding the selection of the materials, it might be helpful to rely on results from randomized controlled clinical trials (RCTs). A recent review by the Cochrane Review Group emphasized that only a few RCTs are available for comparisons between metal-free and metal-based restorations, concluding that there is no evidence regarding the superiority of any of these materials. As a consequence, the authors suggested that dentists should base their decisions on their clinical experience, the individual circumstances, and the opinion of the patient [10]. In comparison to the results presented by the Cochrane Review Group, another research group included a wider range of study designs in a systematic review on the survival and complication rates of ceramic or metal-ceramic single crowns (SC). The authors concluded that SCs fabricated from ceramics showed similar survival rates as PFMs, yet layered zirconia SCs were more prone to technical complications such as chipping of the veneer [2].

In recent years, the National Dental Practice-Based Research Network (PBRN) Collaborative Group has surveyed dentists in the United States (US). Among others, the topics of interest included the frequency of specific dental procedures, general health aspects in dentistry, and questionnaires related to tooth-supported SCs [11–13]. Regarding the selection of materials for fabricating SCs in the anterior area, the study identified that lithium disilicate (54%), layered zirconia (17%), and glass ceramics (13%) were favored by the participating dentists. For posterior SCs, monolithic zirconia (32%), PFM (31%), and lithium disilicate ceramics (21%) were preferred. The authors concluded that the selection of the materials was significantly associated with the individual characteristics of the participating dentists and their patients [12].

In comparison to the US, guidelines of the German national health care insurance companies compile minimum standards for both the type of restoration and the restorative material depending on the individual clinical setting. One of these companies has shared data for scientific purposes over the last years [14], yet only general information can be retrieved from that database. Specific but relevant aspects of prosthetic restorations, such as the type of materials or the individual clinical situation (i.e., marginal preparation design), were not documented in detail. Thus, no valid estimation regarding the preferred materials of dentists in Germany for the fabrication of SCs is possible from that dataset. Moreover, it is unclear whether these preferences depend on the individual characteristics of the dentist, the dental practice, or the patient.

Thus, the aim of this study was to survey dentists in Germany to identify their favored materials for the fabrication of tooth-supported SCs depending on the location of the abutment teeth and the preparation margin. The working hypothesis was that dentists in Germany recommend the same materials for the fabrication of SCs independent of the individual clinical scenario, the characteristics of the dentists, or their dental practice.

## Materials and methods

### Questionnaire design and pretesting of the questionnaire

The questionnaire was developed by a team of three experienced dentists and a statistical data manager. Some features of a survey that had been conducted by the National Dental PBRN investigating the material selection for tooth-supported SCs of US dentists were included [12]. The project was run under the title “Versorgungskompass Festsitzender Zahngetragener Zahnersatz”. Concepts and questionnaire were reviewed by the Executive Board of the German Society for Prosthodontics and Dental Materials Science (DGPro), and revisions were made by the authors.

One part of the questionnaire aimed to gather data regarding the demographic characteristics of the participating dentists, such as their age group, sex, area of expertise, and years since graduation. Another part of the questionnaire focused on characteristics of their dental practice, including the first digit of the postal code and the number of inhabitants in the village/city in which the dental practice/university was located. Moreover, the participants were asked to indicate whether they provided at least one single-unit or multi-unit fixed dental prosthesis/month.

Regarding the preference of materials for the fabrication of SCs, the questionnaire provided four potential locations of abutment teeth, including teeth (according to the FDI) 16, 11, 34, and 36 (A), as well as a description of the individual preparation margin (supra- or subgingival) (B). Each combination of tooth and preparation margin was addressed in a single question (Table 1), resulting in an overall total of eight questions formulated as the following: “What kind of material do you usually recommend for a permanent tooth-supported single crown located on abutment tooth (A) and with a (B) preparation margin?”. Participating dentists were able to choose between full metal, PFM, ceramic, and CAD/CAM resin composite. Moreover, it was possible to give a free-response answer. If ceramics were selected, another question was required, asking the participant to specify the ceramic material from the following: feldspathic/leucite-reinforced glass ceramic, lithium disilicate ceramic, zirconia-reinforced lithium silicate ceramic, monolithic zirconia ceramic, or layered zirconia ceramic. Again, a free-response answer was allowed. Apart from the questions focusing on SCs, the survey included other aspects addressing the material choice for a multi-unit fixed dental prosthesis, cementation, and the intraoral repair of chipping, which will be described elsewhere. The survey was designed to be completed within a maximum time frame of 7 min [15] and was available online (SurveyMonkey, San Mateo, CA, USA) or as a paper-based version provided on demand.

**Table 1:** Structure of the questionnaire investigating the favored materials for the fabrication of single crowns

Sequence	Question	Predefined answers (only one answer allowed)
1	What kind of material do you usually recommend for a permanent tooth-supported single crown located on abutment tooth 16 with a supragingival preparation margin?	• Full metal • PFM • Ceramic • CAD/CAM resin composite • Free response answer:___
2	What kind of material do you usually recommend for a permanent tooth-supported single crown located on abutment tooth 11 with a supragingival preparation margin?	
3	What kind of material do you usually recommend for a permanent tooth-supported single crown located on abutment tooth 34 with a supragingival preparation margin?	
4	What kind of material do you usually recommend for a permanent tooth-supported single crown located on abutment tooth 36 with a subgingival preparation margin?	
5	What kind of material do you usually recommend for a permanent tooth-supported single crown located on abutment tooth 16 with a subgingival preparation margin?	
6	What kind of material do you usually recommend for a permanent tooth-supported single crown located on abutment tooth 11 with a subgingival preparation margin?	
7	What kind of material do you usually recommend for a permanent tooth-supported single crown located on abutment tooth 34 with a subgingival preparation margin?	
8	What kind of material do you usually recommend for a permanent tooth-supported single crown located on abutment tooth 36 with a subgingival preparation margin?	
Additional that occured each time when ceramic was chosen	What kind of ceramic do you recommend (tooth XX, xxgingival preparation margin)?	• Feldspathic/leucite-reinforced glass ceramic • Lithium disilicate ceramic • Zirconia-reinforced lithium silicate ceramic • Monolithic zirconia ceramic • Layered zirconia ceramic • Or material/brand name:__

Tooth location according to the FDI scheme

In August 2018, pretesting of the questionnaire was performed to investigate the practicability of the survey. Ten participants (mean age 34 years, range 26–52 years, 50% female) including both dentists working in private practices (50.0%) and in a university setting (50.0%) were recruited. The think-aloud strategy was used to identify problems while completing the questionnaire. As a result of the pretesting, the coloring of important aspects of the survey, such as the abutment tooth and preparation margin, was revised, and free-response answers were allowed where appropriate (as mentioned in the paragraph above).

### Guidelines and recruitment of participants

Between August 2019 and February 2020, dentists were recruited to voluntarily participate in the survey. In order to reach as many of the 72,592 dentists (45.2% female, mean age = 48.7 years) working in Germany as possible [16], advertisements were placed in various German dental journals, such as *Zahnärztliche Mitteilungen*, *DZZ*, *Quintessenz Deutschland*, or *ZWR*. A newsletter issued by the DGPro was e-mailed several times to invite members of society to participate in the survey. The German Dental Association (BZÄK) supported the survey and asked the chambers of the 16 federal states to display access to the survey on their local websites. Moreover, hand-outs with information about the survey were distributed at various German dental conferences.

Information about data protection was included at the beginning of the survey and could be obtained via file download or paper-based platforms from the authors. The local Ethical Committee approved the investigation (156-19-ek).

### Statistical analyses

Power analyses were based on an overall of 72,592 dentists working in Germany, a margin error of 5%, and a 95% confidence interval and revealed a number of 383 participants. However, to compare differences of 10% between groups by using a power of 90%, a number of 532 participants was determined. The data analysis (SPSS 24, IBM, Armonk, NY, USA) was carried out assuming a significance level of *p* < 0.050. Data were only included if the participants had agreed to provide at least one single crown or multi-unit fixed dental prosthesis (FDP) per month. Besides, dentists who did not work in Germany were excluded prior to statistical analysis. Descriptive data were used to present frequencies and counts of given answers. For group comparisons, the participants who graduated within the last 15 years were placed in a group. Moreover, data of dentists who worked in a village/city counting up to 20,000 inhabitants were summarized. For comparisons of material choice and characteristics of the dentists/dental practice, the following parameters were chosen as references: sex, time since graduation, and the number of inhabitants. Significances were determined using chi-square tests.

## Results

### Demographic data of the dentists participating in the survey

**Fig. 1:**
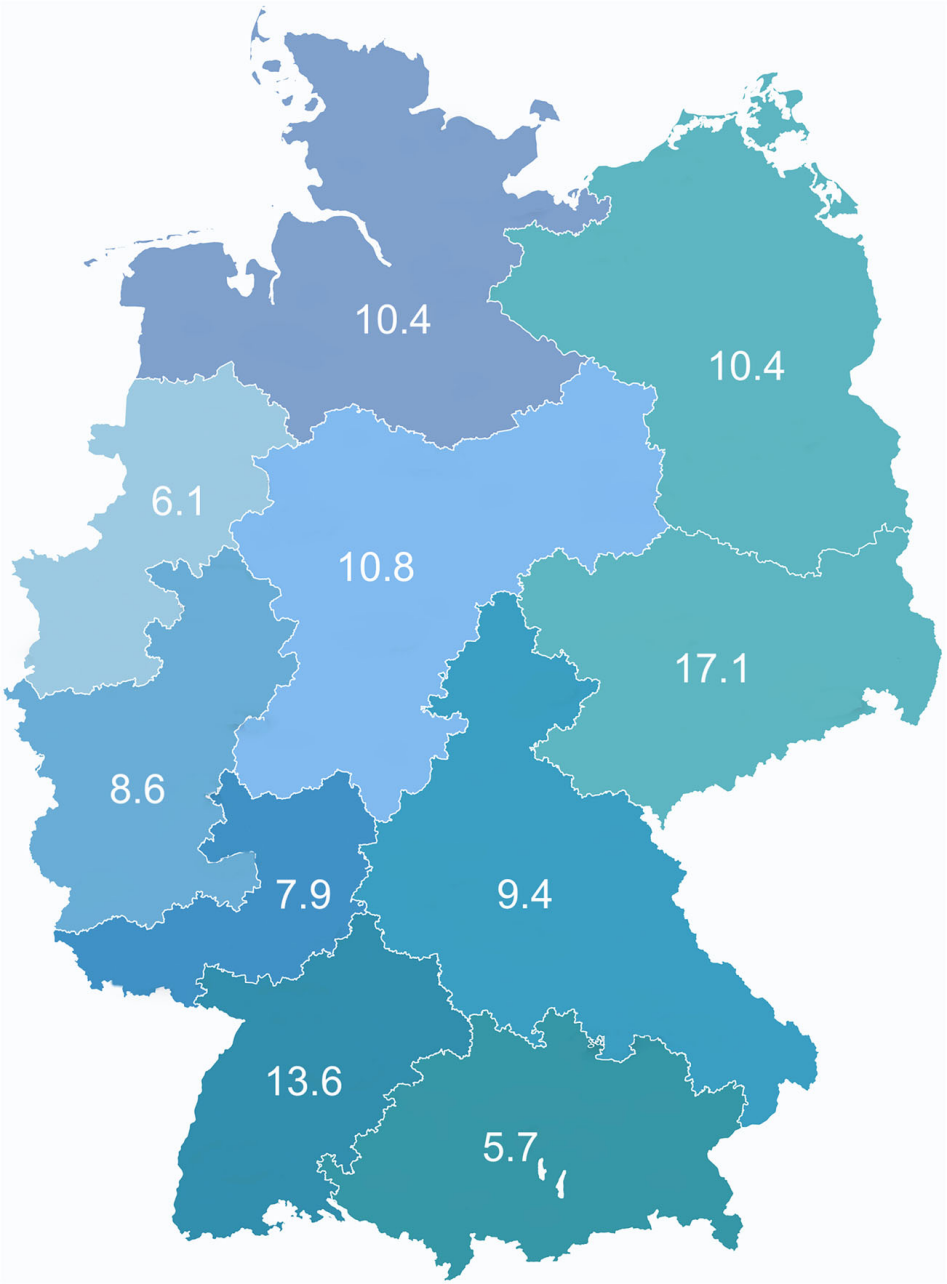
Participation of dentists sorted by postal code as percentage (*N* = 491, no answer = 197)

During a 6-month period, a total of 721 dentists participated in the survey. Six of these 721 dentists stated they were currently not working in Germany, and 27 indicated that they provided less than one single crown or multi-unit FDP per month. The answers of the remaining 688 participants (41.6% female) were included in the data analysis. One third of the participants were younger than 40 years, and approximately 50% were between 40 and 59 years old. According to the postal codes, dentists from all parts of Germany participated in the survey (Fig. 1), and the vast majority declared that they worked predominantly in the fields of conservative or prosthetic dentistry (91.5%). More information on demographic characteristics of the current survey is available in Table 2.

**Table 2:** Information on demographic properties of the participating dentists

	Total	Count	Percentage
Overall	688		
Sex	503		
Female		209	41.6
Male		294	58.4
Age in years	504		
20–29		47	9.3
30–39		142	28.2
40–49		113	22.4
50–59		118	23.4
60+		84	16.7
Years since graduation	503		
< 5 years		59	11.7
5–15 years		160	31.8
> 15 years		284	56.5
Location of dental practice (inhabitants)	501		
< 5000		50	10.0
5000–20,000		122	24.4
20,000–100,000		121	24.2
> 100,000		208	41.5
Area of expertise	403		
Conservative dentistry^a^		204	50.6
Prosthodontics		165	40.9
Others		34	8.5

Missing data due to incomplete questionnaires

^a^Umbrella term for dentists working in the fields of preventive dentistry and cariology

### Materials for SCs with supragingival preparation margins

**Fig. 2:**
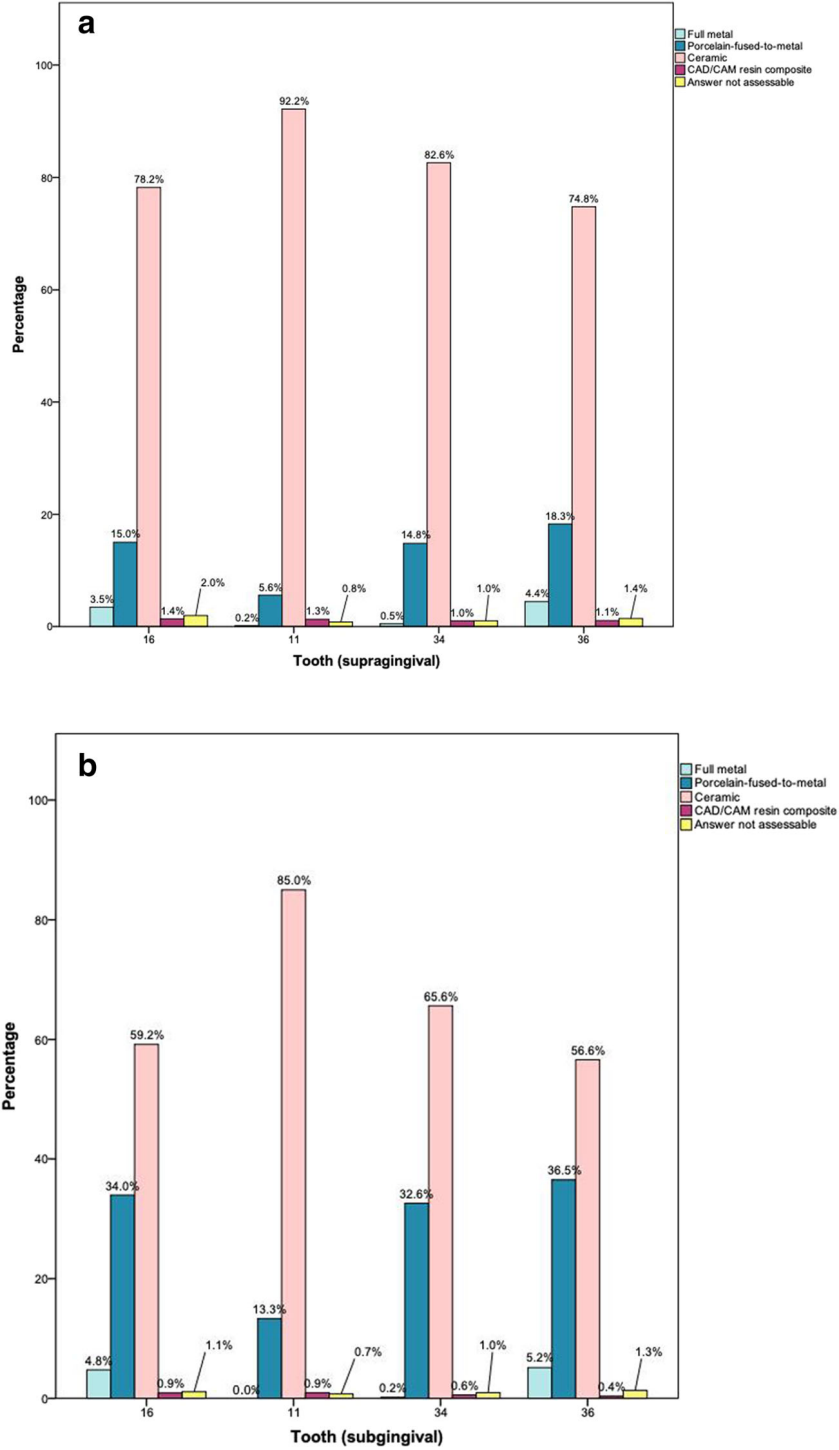
Frequency of favored materials for **a** supragingival and **b** subgingival preparation margins depending on the location of the abutment tooth

Independent of the location of the abutment tooth, ceramic was the treatment option that was most favored by the participating dentists (74.8–92.2%), followed by PFMs (5.6–18.3%) (Tables 3 and 4, Fig. 2a). Full metal was chosen with a frequency of 3.5–4.4% for tooth 16 or tooth 36. The selection of CAD/CAM resin composites as the favorite option reached a maximum of 2.0% for tooth 16. Of those dentists who selected ceramics (Table 5), lithium-X-silicate ceramics were the most favored option (46.0–59.7%) for each of the four abutment teeth, followed by layered zirconia in the anterior area (teeth 11, 34) and monolithic zirconia for molars.

**Table 3:** Frequency in % (count) of answers for single crowns in the upper jaw according to the location of the abutment tooth and the preparation margin in comparison to the sex of the dentist, the years since graduation of the dentist, and the setting of the dental practice

	Supragingival							Subgingival						
	*N*	Full metal	Porcelain fused to metal	Dental ceramic	Indirect composite	Not assessable	*p* value	*N*	Full metal	Porcelain fused to metal	Dental ceramic	Indirect composite	Not assessable	*p* value
Tooth 16														
Overall	665	3.5 (23)	15.0 (100)	78.2 (520)	1.4 (9)	2.0 (13)		544	4.8 (26)	34.0 (185)	59.2 (322)	0.9 (5)	1.1 (6)	< 0.001
Sex														
Male	294	3.7 (11)	16.0 (47)	78.2 (230)	0.7 (2)	1.4 (4)	n.s.	293	4.1 (12)	30.0 (88)	63.8 (187)	1.0 (3)	1.0 (3)	n.s.
Female	209	3.3 (7)	14.8 (31)	79.4 (166)	1.0 (2)	1.4 (3)		209	6.2 (13)	40.2 (84)	51.7 (108)	0.5 (1)	1.4 (3)	
Time since graduation														
≤ 15 years	219	2.7 (6)	13.7 (30)	81.7 (179)	0.9 (2)	0.9 (2)	n.s.	219	4.6 (10)	34.2 (75)	59.8 (131)	0.9 (2)	0.5 (1)	n.s.
> 15 years	284	4.2 (12)	16.9 (48)	76.4 (217)	0.7 (2)	1.8 (5)		283	5.3 (15)	34.3 (97)	58.3 (165)	0.4 (1)	1.8 (5)	
Inhabitants of the village/city in which the dental practice/dental school was located														
< 20,000	172	3.5 (6)	14.0 (24)	80.8 (139)	0.0 (0)	1.8 (3)	n.s.	172	5.2 (9)	32.6 (56)	60.5 (104)	0.0 (0)	1.8 (3)	n.s.
20,000–100,000	121	2.5 (3)	17.4 (21)	78.5 (95)	0.8 (1)	0.8 (1)		120	4.2 (5)	33.3 (40)	60.8 (73)	0.8 (1)	0.8 (1)	
> 100,000	208	3.8 (8)	15.9 (33)	77.4 (161)	1.3 (3)	1.5 (3)		208	4.3 (9)	36.5 (76)	56.7 (118)	1.4 (3)	1.0 (2)	
Tooth 11														
Overall	627	0.2 (1)	5.6 (35)	92.2 (578)	1.3 (8)	0.8 (5)		534	0.0 (0)	13.3 (71)	85.0 (454)	0.9 (5)	0.7 (4)	< 0.001
Sex														
Male	294	0.0 (0)	4.8 (14)	94.6 (278)	0.7 (2)	0.0 (0)	n.s.	294	0.0 (0)	11.9 (35)	86.7 (255)	1.0 (3)	0.3 (1)	n.s.
Female	209	0.0 (0)	5.3 (11)	93.3 (195)	1.0 (2)	0.5 (1)		208	0.0 (0)	14.9 (31)	82.7 (172)	1.0 (2)	1.4 (3)	
Time since graduation														
≤ 15 years	219	0.0 (0)	3.2 (7)	95.9 (210)	0.9 (2)	0.0 (0)	n.s.	219	0.0 (0)	7.3 (16)	91.8 (201)	0.9 (2)	0.0 (0)	0.004
> 15 years	284	0.0 (0)	6.3 (18)	92.6 (263)	0.7 (2)	0.4 (1)		283	0.0 (0)	17.7 (50)	80.2 (227)	0.7 (2)	1.4 (4)	
Inhabitants of the village/city in which the dental practice/dental school was located														
< 20,000	172	0.0 (0)	7.6 (13)	91.9 (158)	0.6 (1)	0.0 (0)	n.s.	172	0.0 (0)	12.8 (22)	84.9 (146)	0.6 (1)	1.8 (3)	n.s.
20,000–100,000	121	0.0 (0)	3.3 (4)	96.7 (117)	0.0 (0)	0.0 (0)		120	0.0 (0)	10.8 (13)	88.3 (106)	0.8 (1)	0.0 (0)	
> 100,000	208	0.0 (0)	3.4 (7)	94.7 (197)	1.4 (3)	0.5 (1)		208	0.0 (0)	14.4 (30)	83.7 (174)	1.4 (3)	0.5 (1)	

Missing data due to incomplete questionnaires

*n.s.* not statistically significant

**Table 4:** Frequency in % (count) of answers for single crowns in the lower jaw according to the location of the abutment tooth and the preparation margin in comparison to the sex of the dentist, the years since graduation of the dentist, and the setting of the dental practice

	Supragingival							Subgingival						
	*N*	Full metal	Porcelain fused to metal	Dental ceramic	Indirect composite	Not assessable	*p* value	*N*	Full metal	Porcelain fused to metal	Dental ceramic	Indirect composite	Not assessable	*p* value
Tooth 34														
Overall	593	0.5 (3)	14.8 (88)	82.6 (490)	1.0 (6)	1.0 (6)		521	0.2 (1)	32.6 (170)	65.6 (342)	0.6 (3)	1.0 (5)	< 0.001
Sex														
Male	294	0.7 (2)	14.6 (43)	83.7 (246)	0.7 (2)	0.3 (1)	n.s.	291	0.3 (1)	27.8 (81)	70.8 (206)	0.3 (1)	0.7 (2)	n.s.
Female	209	0.0 (0)	14.8 (31)	81.8 (171)	1.9 (4)	1.4 (3)		207	0.0 (0)	39.1 (81)	59.4 (123)	0.5 (1)	1.0 (2)	
Time since graduation														
≤ 15 years	219	0.0 (0)	12.8 (28)	85.8 (188)	0.9 (2)	0.5 (1)	n.s.	219	0.0 (0)	32.9 (72)	65.8 (144)	0.9 (2)	0.5 (1)	n.s.
> 15 years	284	0.7 (2)	16.2 (46)	80.6 (229)	1.4 (4)	1.1 (3)		279	0.4 (1)	32.3 (90)	66.3 (185)	0.0 (0)	1.1 (3)	
Inhabitants of the village/city in which the dental practice/dental school was located														
< 20,000	172	0.0 (0)	14.5 (25)	84.9 (146)	0.6 (1)	0.0 (0)	n.s.	170	0.0 (0)	31.2 (53)	68.2 (116)	0.0 (0)	0.6 (1)	n.s.
20,000–100,000	208	0.8 (1)	14.9 (18)	82.6 (100)	0.8 (1)	0.8 (1)		119	0.0 (0)	31.1 (37)	68.1 (81)	0.0 (0)	0.8 (1)	
> 100,000	208	0.5 (1)	14.4 (30)	81.7 (170)	1.9 (4)	1.4 (3)		207	0.5 (1)	33.8 (70)	63.8 (132)	1.0 (2)	1.0 (2)	
Tooth 36														
Overall	563	4.4 (25)	18.3 (103)	74.8 (421)	1.1 (6)	1.4 (8)		523	5.2 (27)	36.5 (191)	56.6 (296)	0.4 (2)	1.3 (7)	< 0.001
Sex														
Male	293	4.8 (14)	16.4 (48)	76.5 (224)	1.0 (3)	1.4 (4)	n.s.	294	4.4 (13)	32.0 (94)	61.9 (182)	0.3 (1)	1.4 (4)	n.s.
Female	209	3.8 (8)	19.6 (41)	73.2 (153)	1.4 (3)	1.9 (4)		208	5.8 (12)	42.3 (88)	50.0 (104)	0.5 (1)	1.4 (3)	
Time since graduation														
≤ 15 years	219	2.3 (5)	16.4 (36)	79.5 (174)	0.9 (2)	0.9 (2)	n.s.	219	3.2 (7)	37.9 (83)	57.5 (126)	0.9 (2)	0.5 (1)	n.s.
> 15 years	283	6.0 (17)	18.7 (53)	71.7 (203)	1.4 (4)	2.1 (6)		283	6.4 (18)	35.0 (99)	56.5 (160)	0.0 (0)	2.1 (6)	
Inhabitants of the village/city in which the dental practice/dental school was located														
< 20,000	172	4.7 (8)	18.6 (32)	75.0 (129)	0.0 (0)	1.7 (3)	n.s.	171	4.7 (8)	34.5 (59)	58.5 (100)	0.0 (0)	2.4 (4)	n.s.
20,000–100,000	120	2.5 (3)	20.0 (24)	75.8 (91)	0.8 (1)	0.8 (1)		121	3.3 (4)	39.7 (48)	56.2 (68)	0.0 (0)	0.8 (1)	
> 100,000	208	4.8 (10)	15.9 (33)	75.5 (157)	2.4 (5)	1.5 (3)		208	5.3 (11)	36.1 (75)	56.7 (118)	1.0 (2)	1.0 (2)	

Missing data due to incomplete questionnaires

*n.s.* not statistically significant

**Table 5:** Frequency in % (count) of answers specifying the dental ceramic according to the location of the abutment tooth and the preparation margin, as well as comparisons of the preferred ceramics for supra- and subgingival preparation margins

Tooth/preparation margin	Total	Classical glass ceramics	Lithium-X-silicate ceramics		Monolithic zirconia	Layered zirconia	Others^a^	Not assessable	*p* value	Porcelain fused to metal (counts, see Tables 3 and 4)
		Feldspathic/Leucite-reinforced	Lithium disilicate	Zirconia-reinforced lithium silicate ceramic						
16 supragingival	495	1.6 (8)	31.1 (149)	21.1 (104)	28.1 (139)	17.8 (88)	0.2 (1)	1.2 (6)	< 0.001	100
16 subgingival	312	1.0 (3)	17.0 (53)	16.3 (51)	41.7 (130)	23.1 (72)	0.3 (1)	0.6 (2)		185
11 supragingival	561	8.6 (48)	46.7 (262)	13.0 (73)	2.0 (11)	28.9 (162)	0.0 (0)	0.9 (5)	< 0.001	35
11 subgingival	446	5.2 (23)	35.0 (156)	13.5 (60)	2.9 (13)	43.0 (192)	0.0 (0)	0.4 (2)		71
34 supragingival	473	3.4 (16)	34.9 (165)	18.0 (85)	15.4 (73)	27.1 (128)	0.2 (1)	1.0 (5)	< 0.001	88
34 subgingival	337	2.4 (8)	22.8 (77)	15.1 (51)	22.0 (74)	36.5 (123)	0.3 (1)	0.9 (3)		170
36 supragingival	413	1.0 (4)	29.8 (123)	16.2 (67)	34.6 (143)	17.7 (73)	0.2 (1)	0.5 (2)	< 0.001	103
36 subgingival	294	1.0 (3)	15.3 (45)	17.3 (51)	42.5 (125)	23.1 (68)	0.2 (1)	0.3 (1)		191

^a^Polymer-infiltrated ceramic-network material given as a free answer

### Materials for SCs with subgingival preparation margins

In a setting with subgingival preparation margins, ceramics were preferred by 59.2–85.0% of the participating dentists (Tables 3 and 4, Fig. 2b), followed by PFM (13.3–36.5%). In molars, full metal SCs were recommended by 4.8–5.2% of the participants. CAD/CAM resin composites were rated as the favored treatment option with a range between 0.7 and 1.1%. Participants who selected ceramics (Table 5) ranked lithium-X-silicate and layered zirconia ceramics as their favored ceramics for anterior teeth and monolithic zirconia for posterior teeth.

### Characteristics of dentists/dental practice, preparation margin, and material choice

The survey revealed that the time since graduation statistically significantly influenced the choice of materials for fabrication of a single crown for tooth 11 with a subgingival preparation margin (Table 3). Dentists who graduated within the last 15 years tended to recommend fewer SCs fabricated from PFM than dentists who graduated more than 15 years ago. For sex-related comparisons, no statistically significant differences in material selection were observed. No statistically significant differences in material selection were identified in group comparisons categorized by the number of inhabitants of the village/city in which the dental practice/university was located.

Dentists more frequently choose PFMs for scenarios involving subgingival preparation margins compared to the material chosen for supragingival preparations (all *p* < 0.001, Tables 3 and 4).

**Fig. 3:**
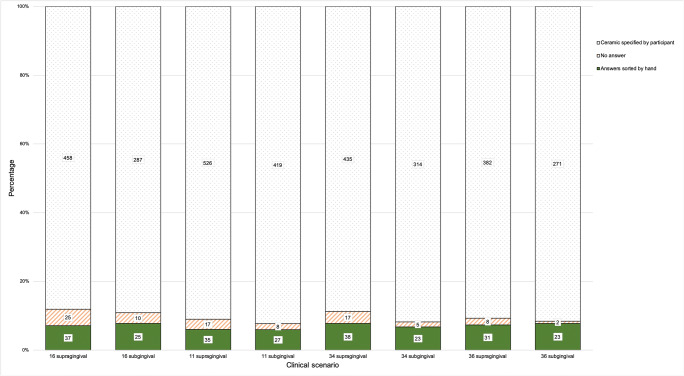
Frequency and count of answers that were given by participants choosing ceramics

Regarding ceramics, 5.7–7.8% of the participants wrote a free-response answer instead of making a choice from the given ceramic classification; up to 4.8% did not specify the ceramic (Fig. 3).

## Discussion

The results of the current survey entitled “Versorgungskompass Festsitzender Zahngetragener Zahnersatz” revealed that ceramic materials are favored by dentists in Germany for the fabrication of SCs, which was independent of the location of the abutment tooth and preparation margin. Only a few participants favored CAD/CAM resin composites or full metals. However, in scenarios with subgingival preparation designs, we observed that participating dentists preferred PFMs more frequently than in settings with supragingival preparation margins. Sex, the time since graduation, and the number of inhabitants of the village/city the dental practice/university was located did not influence the dentists’ preferences except for a single scenario (11 subgingival), which revealed a dependency on the time since graduation. Consequently, the working hypothesis of this investigation can be partially rejected, as characteristics of the participating dentists had—at least for one specific clinical scenario—an impact on the material recommendations.

The data of the current study suggest that the influence on preferences for the material selection of SCs are different between German and US dentists as the latter are more likely to base decisions on their own characteristics or those of their dental practice [12]. Nonetheless, ceramics were favored in both nations, followed by PFMs. The US survey comprised a clinical scenario that also included the fabrication of an SC for abutment tooth 11. The participating US dentists favored lithium disilicate as the restorative material, followed by layered zirconia and classical glass ceramics. In contrast to the US study, the current survey further distinguished between a supra- and subgingival location of the preparation margin. For supragingival preparation margins, the results of our survey were similar to the results from the US as lithium-X-silicate was recommended by most of the participants. However, for the scenario with subgingival preparation margins, the proportion of participants who preferred PFM increased compared to the material of choice for supragingival preparation margins. Both the US and the current survey included a setting that required the selection of an SC material for abutment tooth 36. German dentists recommended lithium-X-silicate ceramics (supragingival preparation margin) and monolithic zirconia, as well as PFM (subgingival preparation margin), whereas US dentists favored predominantly monolithic zirconia, followed by PFM and lithium disilicate. The authors assume that in scenarios in which moisture is difficult to control and clinical access and the view are limited, dentists tend to use materials that can be inserted using conventional cementation methods. Moreover, it might be possible that dentists are not aware or do not rely on the fact that lithium disilicate or zirconia ceramics are approved for conventional cementation techniques in most clinical situations. Although all materials offered in the questionnaire were indicated for SCs in the given location of the abutment tooth or preparation margin, these results also assume that in technically complicated settings, esthetic aspects are estimated to be less important than easy handling and evidence. Nonetheless, the data of the current survey suggest that the application of monolithic zirconia is widely established for molar restorations, although evidence on the long-term performance and survival, as well as that on the effects on the masticatory system, is still sparse.

Surprisingly, some of the participating dentists did not complete the questions about specific ceramics. However, numerous dentists entered a free answer, such as the manufacturer or the trade name. This result might be due to the high fluctuation rates of tooth-colored materials on the dental market [17] and might indicate confusion over existing classifications. Nonetheless, it is important that dentists are informed about the differences between ceramics as this knowledge is relevant when choosing the correct cementation method or indications of the material. These considerations might also explain the few recommendations of CAD/CAM resin composites or polymer-infiltrated ceramic-network materials in the present survey. Regarding their composition and structure, these materials are highly complex and require careful handling in strict accordance with the guidelines issued by the manufacturer. The vast number of different materials that are available on the German market might also produce uncertainties and confusion, which indicates that improved postgraduate education might be required, for instance, by overview articles in the German language or utilizing more detailed curricula.

The limitations of this study include the number of clinical scenarios that were offered to the participants. It might have been interesting to elucidate potential differences in restorative approaches for the lower incisors, equigingival preparation margins, or patients with parafunctional activities. Moreover, it would have been interesting to introduce clinical scenarios with different layer thickness of the SCs as this parameter might also influence the choice of tooth-colored materials. However, the estimated time required for completion of the survey was set to a maximum of 7 min to ensure that a high percentage of participants completed the survey [15]. Nevertheless, the scenarios in the survey covered both esthetically (11, 34) and technically challenging settings (molars). Despite all our efforts, questions were more frequently left uncompleted at the end of the survey. Even though an easy-to-use drop-down menu was employed to answer, e.g., the area of expertise, solely half of the participants completed the question (58.6%).

One percent of the dentists working in Germany responded to the survey, and postal codes indicated a similar percentage of participating dentists from all parts of the country. Moreover, sex- and age-related characteristics of the participants were similar to the overall demographics of dentists in Germany. Nonetheless, for an entirely representative approach, it would have been best to directly address each dentist in Germany, e.g., by e-mail or letter service to ensure that every dentist was invited to take part in the survey and to calculate an actual rate for non-respondents.

The results suggest that dentists selected their restorative materials depending on the individual clinical scenario. Nevertheless, improvements in postgraduate information and education might help to extend the expertise for newly introduced tooth-colored materials.
